# The Conceptual Framework of Dual Disorders and Its Flaws

**DOI:** 10.3390/jcm9072098

**Published:** 2020-07-03

**Authors:** Matteo Pacini, Angelo G. I. Maremmani, Icro Maremmani

**Affiliations:** 1 G. De Lisio Institute of Behavioral Sciences, 56100 Pisa, Italy; paciland@virgilio.it; 2 Department of Psychiatry, North-Western Tuscany Local Health Unit, Tuscany NHS, Versilia Zone, 55049 Viareggio, Italy; angelo.maremmani@uslnordovest.toscana.it; 3 PISA-School of Experimental and Clinical Psychiatry, 56100 Pisa, Italy; 4 Association for the Application of Neuroscientific Knowledge to Social Aims (AU-CNS), 55045 Pietrasanta, Lucca, Italy; 5 Vincent P. Dole Dual Disorder Unit, 2nd Psychiatric Unit, Santa Chiara University Hospital, University of Pisa, 56100 Pisa, Italy

**Keywords:** dual disorders, flaws, conceptual framework

## Abstract

When psychiatric illness and substance use disorder coexist, the clinical approach to the patient is, unsurprisingly, awkward. This fact is due to a cultural context and, more directly, to the patient’s psychiatric condition and addiction behaviors—a situation that does not favor a scientific approach. In dual disorder facilities, several types of professionals work together: counselors, social workers, psychologists, and psychiatrists. Treatment approaches vary from one service to another and even within the same service. It is crucial to provide dual disorder patients with multiple treatments, comprising hospitalization, rehabilitative and residential programs, case management, and counselling. Still, when treating dual disorder (DD) heroin use disorder (HUD) patients, it is advisable to follow a hierarchical algorithm. First, we must deal with addiction: by detoxification, whenever possible. This means starting most patients on anti-craving pharmacological maintenance, though aversion therapy may be appropriate for a few of them. Opiate antagonists may be used with heroin-addicted patients as long as those patients are only mildly ill. In contrast, agonist opioid medications, i.e., buprenorphine and methadone suit moderately and severely ill patients, respectively. Achieving control of mood instability or psychotic episodes is the next step, to be followed by a prevention strategy to counteract residual cravings and dominate mood disorders or psychotic episodes through long-term pharmacological maintenance that is focused on a double target.

Many different terms have been introduced to define the co-occurrence of a psychiatric disease and a substance use disorder. In the recent past, acronyms such as MICA (Mentally Ill Chemical Abusers or Mentally Ill Chemically Affected), MISA (Mentally Ill Substance Abusers), CAMI (Chemical Abuse and Mental Illness), and SAMI (Substance Abuse and Mental Illness) have been used [1]. Other innovations include ‘Dual Diagnosis’ and ‘Concurrent Disorders’ [2,3,4,5,6]. Currently, dual disorder (DD) is the term applied by the World Association on Dual Disorders to people who have an addictive disorder alongside a co-occurring mental one [7].

## 1. Dead Ends and Start Lines in Dual Disorder

Several studies or reviews have discussed the issue of the disease chronology of dual disorder. In other words, how can primary psychiatric disorders be distinguished from substance-induced transient or persistent disorders with similar symptoms? A DSM-based classification is of little help, since the exclusion of putative substance-induced disorders from a primary psychiatric category resulted in little attention being paid to these secondary disorders. Substance-induced disorders are, in fact, commonly regarded as difficult to handle, resistant to treatment, and are without any standard treatment algorithm. Similarly, it is not exactly clear what benefits can be gained by pressing the issue of whether a cluster of symptoms is substance induced or primary. Although it is true that the disorder may manifest itself later, the opposite hypothesis is true too. It is also important to carefully consider whether a cluster of substance-induced symptoms, like some psychotropic medications, can be contraindicated (as when antidepressants trigger bipolar episodes).

In any case, we continue to believe that this issue is a dead end for the following reasons. First, the assumption that substance-induced disorders are different from spontaneous ones is gratuitous. As far as we know, certain substance-induced psychiatric disorders may just be phenocopies of spontaneous versions of the same biological disorder. Moreover, the time overlap between onset periods may lead to overrating of the effect of a substance on the development of certain psychiatric disorders. These disorders would find a way to emerge, whether after a time interval or else gradually over time, in some cases showing a sharp profile of classic symptoms rather than a substance-filtered clinical picture. Lastly, the diagnosis of a “secondary” disorder does not automatically imply that detachment from the substance will lead to a stable extinction of symptoms. On the other hand, the persistence of symptoms long after detachment from substance use and the accomplishment of detoxification do not necessarily indicate a primary disorder. The emergence of psychiatric symptoms after the end of agonist opioid treatment may indicate a therapeutic effect of that kind of treatment on an independent psychiatric disorder, as in the case of methadone-withdrawal psychoses. These disorders are often hard to recognize during agonist treatment. They may be considered as transient withdrawal-related accidents rather than primary disorders that were initially masked within an illustration of severe chronic intoxication and then disappeared during anti-craving treatment.

Psychiatric disorders are heterogeneous, as they share no common source for all psychiatric symptoms. Instead of splitting a patient population into sharply defined clinical disorders (for instance, affective psychosis and methamphetamine use or panic disorder and alcoholism), most studies prefer to refer to obscure “all-in” categories, such as dual diagnosis, dual disorders, psychiatric comorbidity, and “psychiatric symptoms.” Moreover, no distinction is drawn between different substance classes. The result is that we are forced to reason over treatment approaches to methamphetamine addicts with psychotic symptoms who are lumped together with opioid addicts, who have to cope with depression, or alcoholics, who have to contend with social phobia. We agree on the usefulness of a research “field” that resorts to grouping together all such conditions, as long as there is a common ground of clinical and biological knowledge. In general, research projects would achieve more if they were based on clearly defined targets and study populations.

Substance use disorders are heterogeneous, too, since not all clinical symptoms correspond to a chronic relapsing loss of control over use (i.e., addiction). Also, not all cases of poly-use have the same dynamics concerning primary addiction and any concurrent psychiatric disorder(s). In greater detail, we assessed patients with alcohol–heroin poly-use, cocaine–alcohol poly-use, and heroin–cocaine poly-use, comparing them with exclusive users of heroin, alcohol, and again heroin, respectively. In some cases, other minor substances, mostly cannabis and benzodiazepines, were involved too, even if their use had not been the original reason for treatment. Studies have agreed on indicating cocaine abuse as correlated with axis I bipolar disorder, whether it is combined with alcohol or heroin. On the other hand, the heroin–alcohol poly-use pattern is typical of highly cyclothymic subjects, whose data, however, remain below the threshold of clinical diagnosis [8,9,10,11,12]. Depressive disorders are unrelated to either combination. The heroin–alcohol combination often develops because of treatment omission, premature termination, or, simply, undermedication, so that it seems at first glance to be a surrogate or enhanced form of a common opioid-based drug disorder [13,14]. The cyclothymic profile is, in fact, the only profile that discriminates heroin- and alcohol-dependent patients from healthy controls [15,16].

Apart from addiction-centered studies, other authors have indicated stimulant use, possibly coupled with alcohol and cannabis, as peculiar to a bipolar diathesis. These authors proposed the concept of bipolar-stimulant spectrum disorders, going beyond the causal distinction between spontaneous, associated, and induced bipolar disorders [17].

## 2. Screening and Definition Criteria for Dual Disorder Heroin Addiction

In several studies, no psychiatric category is specified when reporting on the comorbidities of substance abusers; instead, authors refer to comorbid mental disturbance by drawing on syndrome names as labels or naming series of key symptoms. The foremost criterion for the screening of dual disorders should be the deviance of putative diagnosis from the stereotype of transient chronic intoxication, either during substance use or soon after detoxification. Such a stereotype varies according to which substance is accounted for, even if not defined during mixed poly-use phases. Nevertheless, the stereotype of heroin addiction has been reliably defined as the depressive–anxious–hypersensitive–somatic syndrome. This clinical picture runs parallel to acute opioid impairment (susceptibility to withdrawal) and the severity of the addiction. A variant of the same syndrome can be identified as the hypophoric syndrome (“reward deficit” syndrome) [18] following detachment from opiates or agonist treatment subtraction, otherwise known as the late withdrawal syndrome. This latter condition is well known to be an indicator of relapse, sensitive to opiate agonists, and worsened by antagonists. Italian authors have worked to ascertain the exact reasons why the above conditions should not be labeled as dual disorders or at least are not enough to authorize the recognition of an independent mood or anxiety disorder.

On the other hand, psychotic states, as well as substance-related excitement, are quite unlikely during opiate maintenance, even in patients who may be abusing cocaine during methadone maintenance [19].

European data on the prevalence of psychosis in AOT populations show a relatively low rate of schizophrenia or delusional disorder, regardless of the rate of global comorbidity. In the Netherlands, as many as 39% of opioid users in treatment do display psychotic symptoms, out of an 84% overall comorbidity rate, but current (acute) psychosis does not reach the 10% level. A small population Italian study on hospitalized substance abusers (heroin being featured as the primary substance) described the effects of methadone dose increase and the reduction of antipsychotics and mood stabilizers at discharge, under similar conditions for the length of stay in hospital and the kind of index diagnosis at admission [20]. On the whole, the current evidence favors the view that opioid agonist treatments have a therapeutic influence on psychotic states (an influence that is dependent on the doses being used), and that this link may mask the prevalence of psychotic disorders in populations maintained on over-standard doses.

## 3. Dual Disorder Patients and Treatment System

The medical approach to the dual disorder (DD) patient is inevitably awkward. This fact is due to a cultural context and involves both the patient’s psychiatric condition and addiction behaviors. This predicament does not facilitate a scientific approach to psychiatric illness, in general, or, more forcefully, to addictive diseases. On the one hand, depression and worries about effectiveness are unlikely to restrain patients from resorting to medical services. On the other hand, environmental issues interfere with the correct treatment system. Patients are unlikely to know what kind of treatment is provided by which service. Some services, though effective, are only available in some areas, while others are only available if paid for; therefore, those addicts in some parts of a country have difficult issues to face.

When DD patients apply to an outpatient clinic to receive treatment for their addiction, it often happens that acute psychiatric disorders are misdiagnosed for substance-induced ones or, conversely, intoxication or withdrawal symptoms are misinterpreted as psychiatric illness. In the latter case, patients are usually transferred to psychiatric services. Paradoxically, the same happens with psychiatric patients who apply for treatment at psychiatric units, if they are also current substance use disorder (SUD) patients [21]. The frequency and intensity of psychiatric symptoms and substance-induced symptoms usually rise and fall. So, the need to buffer irregular acute variations on a basis comprising a dual disorder may catch the clinician’s attention more than the need to control the independent aspects of the case, which will be addictive, psychiatric, and social. The result may be that the national health system becomes an impediment to patients seeking treatment, rather than a manner of offering them adequate health facilities. Currently, a correct approach to DD patients requires not only that attention be dedicated to the specific issues of each patient but also calls for a growing awareness of the continuing discrepancy between the health system, as it is, at present, implemented, and the needs of DD patients.

Several types of operators work together in psychiatric services: counselors, social workers, psychologists, psychiatrists, and others, on a case-by-case basis. Treatment strategies vary between services and within the same service. It is crucial to provide psychiatric patients with integrated treatments, comprising hospitalization, rehabilitative and residential programs, case management, and counseling, to satisfy the needs arising from both acute and chronic conditions. In some cases, psychotropic medications are used to treat psychiatric disorders and SUD at the same time. The frequency of use of nonmedical psychotropics in treating general psychiatric patients is low, whereas DD patients tend to abuse otherwise harmless agents, such as sedative tricyclic antidepressants [22,23] and antipsychotics [24,25,26,27,28,29,30,31,32,33]. Problems may, therefore, follow from the incautious prescription of psychotropic medication to addiction-prone patients. So, psychiatrists should extend their knowledge of substance-related medical issues, while physicians treating drug addicts should take the trouble to become knowledgeable about psychiatry, especially the use of psychotropic medications. As in general psychiatry, a variety of therapeutic solutions are available for the treatment of SUD patients. We can list agonist maintenance, therapeutic communities, short- and long-term detoxification programs, and self-help programs, which often utilize divergent basic principles and may be discordant with each other. Some programs require a patient’s drug-free condition as indispensable to initiating treatment, whereas becoming drug-free is simply the long-term result of other programs. Methadone or buprenorphine maintenance therapy does not invariably aim at the complete elimination of heroin use. Controlled heroin use may be adequate, when no other programs are successful, as long as methadone maintenance ensures satisfactory personal and social recovery. The coverage of heroin-assisted treatment (HAT) in countries where it is available is modest when compared with other agonist maintenance treatments for heroin use disorder (HUD). Within the European Union, the role of HAT is negligible. A range of therapeutic, prevention, safety, and economic concerns about the possible negative effects of HAT, for patients and the treatment system, are debated in the light of pertinent research evidence [34,35,36,37,38,39]. None of these concerns are justified. Encouraging effects should predominate on the treatment system and public order. The HAT methodology has good outcomes for previously treatment-resistant HUD patients, besides deserving consideration as a safe, useful element in a comprehensive treatment system for HUD patients and, crucially, a cost-effective therapy [40].

Although the teams working in addiction medicine units comprise counselors, psychologists, psychiatrists, and physicians, other professionals may be involved too, offering a variety of ancillary skills. The integration, according to a biopsychosocial approach, of various professional skills, should be placed at the core of any service directed to combating addiction. Psychotropic medications are currently used to treat overdose and withdrawal symptoms in SUD patients, but some of these medications, especially disulfiram, naltrexone, and methadone, are effective on addiction too. Addiction controlling physicians are often knowledgeable about psychiatric medications, but a prejudice exists that any psychotropic medication is expected to induce dependence. Especially in countries with separate psychiatric and addiction units, many addiction physicians avoid prescribing psychotropic medications, whereas they should be skilled and knowledgeable in resorting to them and being able to choose the right type for specific psychopathological conditions. In this kind of service, unless DD patients are supported with effective treatment for their mental illness, the risk of relapse is destined to remain high.

Alcoholics Anonymous and Narcotics Anonymous—two types of self-help associations—may have much to offer to DD patients. Self-help interventions should not be considered as alternative treatment options but be integrated into a comprehensive treatment. On the other hand, speculative fears and misinformation may spread within self-help groups, if participants never go beyond reporting opinions based on personal experiences. In the United States, specific self-help programs for DD patients have been developed by focusing on the advancement of patients’ compliance with psychopharmacological treatment.

DD patients commonly get in touch with their GPs, but they regularly get only minor attention. In Italy, e.g., GPs are likely to deal with DD patients by prescribing generic psychotropics, such as anxiolytics and antidepressants, but not abuse-targeting medications, such as disulfiram and naltrexone, whose use is limited to specialized programs. GPs are the category of physician that is most prone to prescribe benzodiazepines as anxiolytic drugs, although benzodiazepines carry the highest risk of nonmedical use. Generally speaking, GPs are most concerned about the complications of addiction, such as overdosing, withdrawal symptoms, or physical issues, rather than aiming for an intervention that targets the core of the addiction. There are only a few cases in which GPs are involved in the treatment of DD patients [41,42,43,44]

## 4. Case Management of Dual Disorder Patients

The public health system has constantly given patients the responsibility of presenting for treatment as a sign of being motivated to ask for treatment. More recently, the same issue has been introduced with reference to what is called “case management” (CM). Most DD patients are, in fact, reluctant to resort to local addiction units or are unable to benefit from available facilities. CM may be a crucial resource for SUD patients when the aim is to include patients in treatment programs and improve their retention in treatment. CM may also help attenuate the negative results of leaving treatment. Conversely, programs without a CM are more likely to be handicapped by hospitalization episodes and psychopathological crises, while the most complicated cases are unlikely to be successfully resolved. The main aim of CM is to encourage hesitant patients to request treatments and limit the negative impact of treatment breakdowns on the personal history of those patients. DD patients need to be followed up for both their conditions, addictive and psychiatric, by applying strategies formulated to fit the specific details of their condition. Patients as well as physicians should make a contribution to treatment. At present, physicians treat patients, who, in responding, tend to deny the presence or minimize the severity of their condition, often with excessive emphasis. DD patients require a very different method in accepting and complying with the treatment. It is prudent to avoid confrontation with particularly severe patients, such as psychotic ones, because they are unable to comply with the rules of the program until the severity of their condition has shown considerable improvement. Too often, addiction is regarded with a “here and now” attitude by physicians, who also tend to exaggerate the environmental aspects of co-present psychiatric disorders. SUD tends to be considered as symptomatic of previous psychic trauma, rather than having the status of an independent illness. Too often, treatment focuses on the psychotherapeutic resolution of some developmental age problem, in the mistaken supposition that addiction will remit once its background issues have been resolved. So far, the main outcome of this bias has been a perpetuation of the vicious circle of addiction.

Some treatment programs require patients to be drug free as a precondition for entering treatment. In most DD patients (such as people with schizophrenia), a drug-free state should only be considered as a possible long-term outcome of enhanced methadone maintenance. On the other hand, a drug-free condition may be useful for patients with depression or panic disorder, to allow an earlier, correct diagnosis and, later, more adequate treatment. For DD patients, imposing a preliminary drug-free condition to allow entry into treatment actually turns out to be an obstacle [45]. We, therefore, suggest that the concept of a “drug-free state” be redefined as a therapeutic goal to be approached step-by-step in parallel with an adequate treatment program. Homeless patients who dwell in highly drug-polluted environments cannot be expected to reach a drug-free condition after the imposition of a hard-and-fast deadline.

## 5. Towards a Hierarchical Approach to Dual Disorder Treatment

We consider three types of treatment models, i.e., sequential, parallel, and integrated, and we propose our hierarchical approach.

The sequential model has been the first one to be employed, and, up to the present time, has been the most frequently utilized. According to this model, the psychiatric and the addictive dimensions of the DD are approached in two different stages. Some clinicians reckon that the addiction should always be approached first and that it is possible to treat the comorbid psychiatric syndrome once any nonmedical use has ceased. Others claim that specific treatments for the psychiatric part of DD may be feasible even when there is the ongoing use of a substance before any specific intervention is taken to end addiction. Another view is that the decision on treatment priority should consider the severity of each condition, with precedence being given to the condition most urgently calling for treatment. For example, we could consider the case of a DD depressed HUD patient requesting treatment at a psychiatric clinic when still suffering from depression, while also participating in a specific program to cure his recurrent alcohol binges.

In the parallel model, the patient is enrolled in two programs simultaneously, the first treating the psychiatric part and the second focusing on addiction. A twelve-step program, may, for instance, be associated with psychiatric treatment under the supervision of mental health professionals. As with the sequential model, this model too consists of a combination of already ongoing programs. Psychiatrists deal with the psychiatric illness, while addiction physicians manage the addiction-related problems. The integrated model combines psychiatric and addiction treatments in a single program, which has been specifically tailored to meet the needs of DD patients. Theoretically, two distinct categories of physicians and skills should be involved, together with a twofold CM approach. This would allow patients to avoid being overwhelmed by the double danger of psychiatric and addictive relapses. Each of these treatment models has its pros and cons. Requirements for treatment adequacy vary with different states of comorbidity, symptom severity, and global functional impairment. The sequential and parallel models may best fit severely addicted patients who also suffer from a minor psychiatric disease. The main negative aspect of these approaches is that patients may receive contradictory information in the two different settings. By contrast, in our opinion, when a CM facility is available and is expressed through a single operator possessing the two sets of skills, which are appropriate to that specific setting, patients would get the full benefits of a binary, two-edged treatment approach [46].

The integrated model is an advanced one. Criteria have even been proposed to determine what constitutes an integrated treatment [47], with preliminary meta-analyses attesting to its efficacy beyond nonintegrated treatments [48].

In our opinion, when treating DD/HUD patients, it is advisable to follow a hierarchical algorithm [49]. According to our clinical experience, the addiction dimension should be dealt with first, by detoxifying patients, and, certainly, by starting most patients on anti-craving agonist maintenance. It should, in any event, be borne in mind that aversion therapy (e.g., disulfiram) may produce a good outcome for a few patients [50]. Opiate antagonists may be administered to HUD patients as long as those patients are only mildly ill, whereas agonist medications, i.e., buprenorphine and methadone suit moderately and severely ill patients, respectively [51,52]. Achieving control of mood instability or psychotic episodes is the next step. It should, eventually, be followed by a preventive strategy to counter residual cravings and breakthrough episodes of mood disorders or psychotic episodes by using long-term pharmacological maintenance with a double target [53,54]. Relapse prevention must never be understood as complete extinction, but as a trend towards a lower grade of severity, a reduction in frequency, while successfully delaying the possible occurrence of a relapse [55,56]. HUD patients should be considered as a population in which it is possible to register and study the effects of chronic opioid injury and its consequent opioid dysfunctions. The predated body of pharmacological knowledge about the psychiatric properties of methadone and buprenorphine seems to corroborate what emerged from the description of agonist opiate-treated DD/HUD patients by our research group [53,57,58,59,60,61,62]. The toxic properties of fast-acting opiates and the therapeutic properties of slow-acting ones prove to be crucial issues in HUD patients, whether they do or do not have DD. Methadone and buprenorphine should be recognized as psychoactive medications, with useful properties in the treatment of opiate addiction, having a wider therapeutic potential when heroin addiction is combined with a psychiatric disorder [20,49,57,59,61,63,64,65,66,67,68,69,70,71,72,73].

## 6. Conclusions

To sum up, dual disorders may be present in cases of intense affective discomfort, especially when patients are free from current intoxication or are emerging after a long period of well-being after discharge from opiate agonist treatment. In all other cases, an addiction-related profile should be considered first—a profile that is likely to be improved by opiate agonist initiation, dose increase, or reintroduction. Psychotic symptoms are more likely to indicate a dual disorder as being responsible for psychosis, except in situations of enforced acute withdrawal or acute psychotomimetic intoxication [74].

The intermingling between substance abuse and psychiatric risk disposition, or primary milder disorders, may lead to full-blown syndromes which would not have developed spontaneously, but do so because of exposure to at least one substance. In such cases, it is not always possible to ascertain the course of the associated disorder, especially when anti-craving therapies are used, which may have a dual effect. Otherwise, the course of the disorder in the absence of relapse will help to bring clarification. The latest clinical configuration should be accounted for the symptoms. For instance, a bipolar type 2 disorder ranking up to type 1 after substance abuse should be rated as bipolar 1. In any case, the course of the condition is expected to be more favorable in a substance-free condition [75].
